# Description of a new species of *Potamonautes* MacLeay, 1838, from the iSimangaliso Wetland Park, South Africa

**DOI:** 10.3897/zookeys.503.9532

**Published:** 2015-05-11

**Authors:** Nasreen Peer, Renzo Perissinotto, Gavin Gouws, Nelson A.F. Miranda

**Affiliations:** 1 DST/NRF Research Chair in Shallow Water Ecosystems, Nelson Mandela Metropolitan University, PO Box 77000, Port Elizabeth 6031, South Africa; 2 South African Institute for Aquatic Biodiversity (SAIAB), Private Bag 1015, Grahamstown, 6140, South Africa

**Keywords:** Brachyura, freshwater, *Potamonautes*, taxonomy, ephemeral pans, sand forest, iSimangaliso Wetland Park

## Abstract

A new species of freshwater crab, *Potamonautes isimangaliso* **sp. n.**, is described from the western shores of False Bay, Hluhluwe, within the iSimangaliso Wetland Park, South Africa. While bearing a superficial resemblance to *Potamonautes lividus*, the new species has been found to be genetically distinct, diverging from the former by 7.4–7.8% in mtDNA. *Potamonautes isimangaliso* most closely resembles *Potamonautes lividus*, but is distinguished by a unique suite of carapace characters, colouration, and size. The new species also lives in close association with oxygen-poor, fresh ephemeral pans, while the habitat of *Potamonautes lividus* is well above the surface water line of the closest water body. An updated identification key for the *Potamonautes* species of South Africa is provided.

## Introduction

Freshwater crabs play a key role in ecosystems by serving as an important food source for larger taxa, and recycling nutrients through detritivorous feeding habits (Cumberlidge 2009). They link terrestrial and aquatic habitats by moving between the two systems, and are considered bioindicator species of environmental change in some habitats (Schuwerack et al. 2001). *Potamonautes* is the only genus of primary freshwater crab (Yeo et al. 2014) in South Africa, with 16 described species occurring in the country, two having been described in recent years (Daniels and Bayliss 2012; Phiri and Daniels 2014).

**Figure 1.**
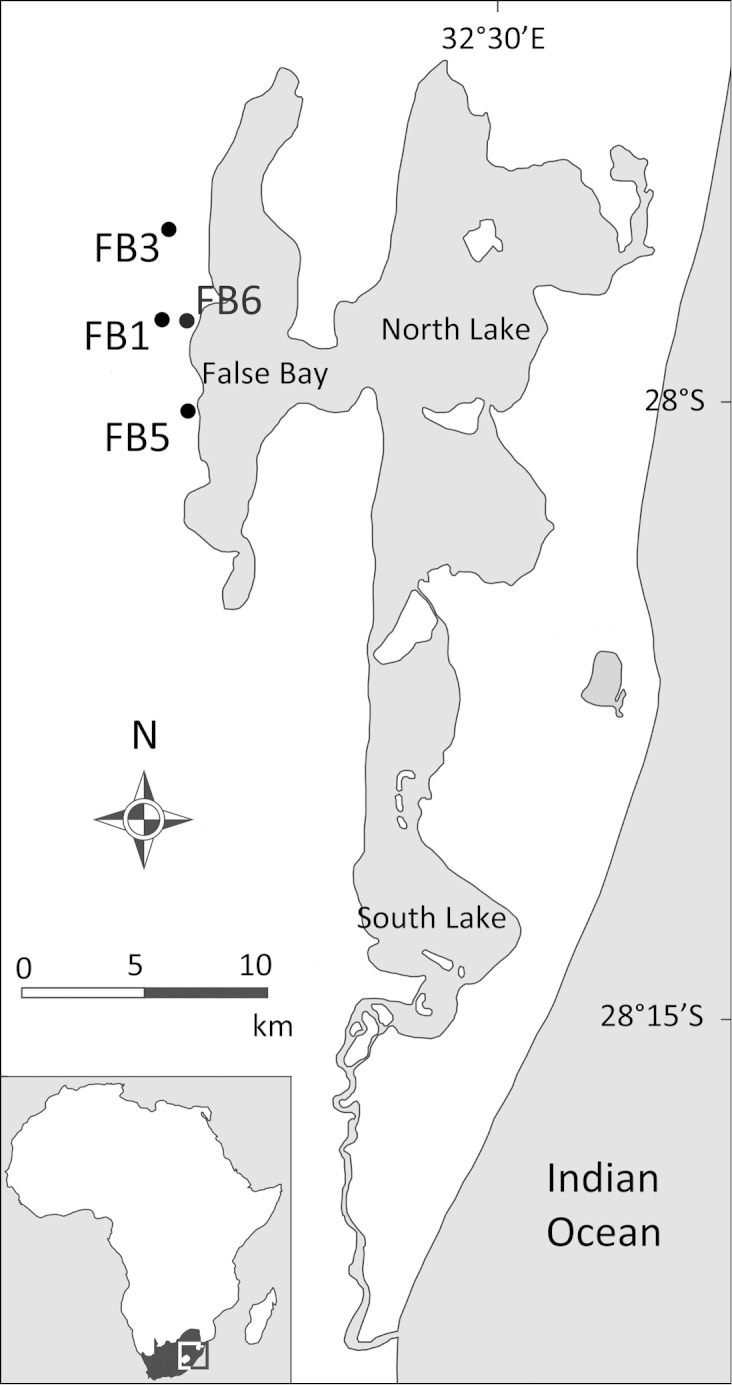
Map of Lake St Lucia on the east coast of South Africa. Collection localities, indicated by black dots and labelled with codes, are all restricted to the western shore of False Bay, within the False Bay Park. FB1 = Main Road Pan; FB3 = Mpophomeni Pan; FB5 = Dukandlovu Pan; FB6 = Sandy Point Pan.

The iSimangaliso Wetland Park forms the southernmost region of the Maputaland centre of endemism and constitutes the focus of biodiversity conservation in the region (Smith et al. 2008). Recent ecological and biodiversity surveys of Lake St Lucia have been conducted in an attempt to update local taxonomic records, identify undescribed species, highlight the change in diversity over time, and provide illustrated and annotated checklists to use as identification tools (Nel et al. 2012; Peer et al. 2014; Perissinotto et al. 2014). Surveys have revealed the existence of an undescribed species of *Potamonautes* along the western shores of False Bay (Fig. 1) that most closely resembles *Potamonautes lividus* Gouws, Stewart & Reavell, 2001 in morphological appearance, exhibiting a rounded vaulted carapace and the potential ability to spend a large amount of time out of water. However, genetic analysis showing a 7.4–7.8% difference across the combined 16S rDNA and COI gene fragments in comparison to *Potamonautes lividus* (G. Gouws unpubl.) and morphological analysis (present study) indicate that the two species are indeed distinct.

In this paper we describe *Potamonautes isimangaliso* sp. n. from the sand forests of the iSimangaliso Wetland Park. NP and GG wrote the taxonomic part of this study, including the description of the new species, while the contribution of the other authors dealt with natural history and ecological observations.

## Materials and methods

### Collection of crabs

Crabs were collected from four localities (Fig. 1) using a sweep net in pans or by active hand capture. The unidentified species was found in 2012–2013 during routine surveys in the area as part of an ongoing project on the biodiversity of Lake St Lucia, supported by the iSimangaliso Park Authority and the provincial conservation authority, Ezemvelo KZN Wildlife, and subsequently during a dedicated survey undertaken in February 2015. Specimens were preserved in 10% formalin or 70% ethanol, once photographs were taken using a Canon Powershot G12.

### Morphological and morphometric analyses

In the laboratory, a pair of Vernier callipers was used to measure morphological variables. A Nikon SMZ25 microscope fitted with a Nikon Digital Sight DS-Fi2 camera was used for macro-examination and to take photos of gonopods and mouthparts.

Abbreviations for depositories and provinces:

ISAM Iziko South African Museum, Cape Town, South Africa

NMMU Nelson Mandela Metropolitan University Collection.

EC Eastern Cape

WC Western Cape

KZN KwaZulu–Natal

Abbreviations for all morphological and morphometric characters (Gouws et al. 2001)

CL Carapace length

CWW Carapace widest width

CWP Carapace posterior width

PFCD Distance between postfrontal crest and anterior margin

ED Distance between orbits

CWA Distance between exorbital teeth

CH Carapace height

AW6 Width of sixth abdominal segment

MCPL Major cheliped propodus length

MCPH Major cheliped propodus height

P2**ML** Pereopod 2, merus length

P2**MH** Pereopod 2, merus height

s2/s3 First sternal groove (suture between the second and third sulci)

s3/s4 Second sternal groove (suture between the third and fourth sulci)

CRDL Right cheliped, dactyl length

CLDL Left cheliped, dactyl length

CRPL Right cheliped, propodus length

CLPL Left cheliped, propodus length

CRPW Right cheliped, propodus width

CLPW Left cheliped, propodus width

ML Merus length

MW Merus width

For the morphometric analyses, eight variables (CL, PFCD, CWP, ED, CH, AW6, CRPL and CRPW) were log transformed and used to run a stepwise discriminant function analysis in STATISTICA v 12.5 (Statsoft 2004). Data for *Potamonautes lividus* were obtained from G. Gouws and represent the specimens used for the description of *Potamonautes lividus* (Gouws et al. 2001). Classification functions were calculated and individuals were then reassigned to groups based on a priori probabilities. Canonical scores were plotted for both species on a frequency histogram to support distinction between the two forms. Lastly, a linear regression analysis was used to examine variation for specific variables.

### Genetic analysis

DNA was extracted from each specimen and amplification of the mitochondrial cytochrome c oxidase subunit I (COI) and 16S ribosomal DNA genes were carried out following protocols outlined by G. Gouws (unpubl.). Amplifications were confirmed by electrophoresis in 1% agarose gels with an ethidium bromide stain. The product was then viewed on an ultraviolet transilluminator. Sequences were generated from a representative of the new species, using approaches described elsewhere (Daniels et al. 2002; Phiri and Daniels 2014; G. Gouws (unpubl.)).

## Results

### Morphometric analysis

**Figure 2.**
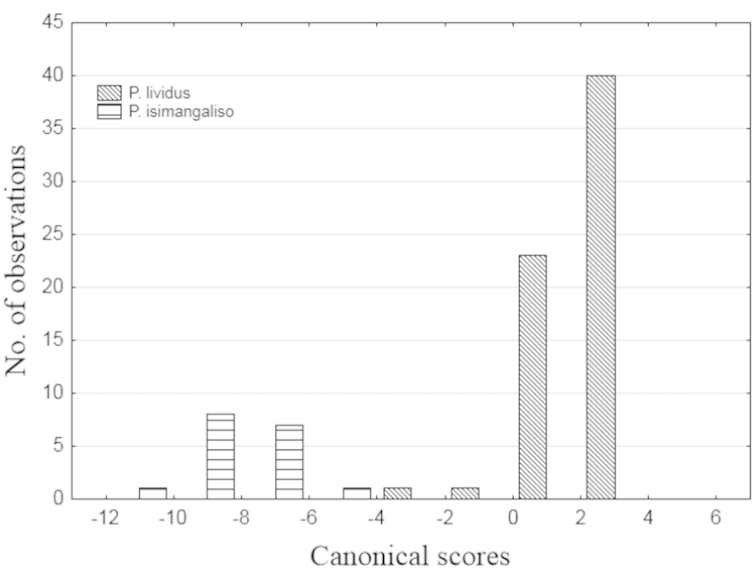
Histogram of canonical scores for *Potamonautes isimangaliso* and *Potamonautes lividus* calculated from a discriminant function analysis.

The new species (*Potamonautes isimangaliso* sp. n.) was distinguished from *Potamonautes lividus* by its larger size, flatter carapace and more rounded posterior. The carapace variables CL, CH and CWP contributed the most to distinguishing between the two forms in the discriminant analysis. Fig. 2 highlights the morphometric distinction between the two species.

The classification function was calculated for both species as follows:

Y(*Potamonautes isimangaliso*) = 926.798(LogCL) – 602.076(LogCH) – 7.966(LogCWP) – 178.319

and

Y(*Potamonautes lividus*) = 1428.33(LogCL) – 743.234(LogCH) – 321.805(LogCWP) – 296.179

**Figure 3.**
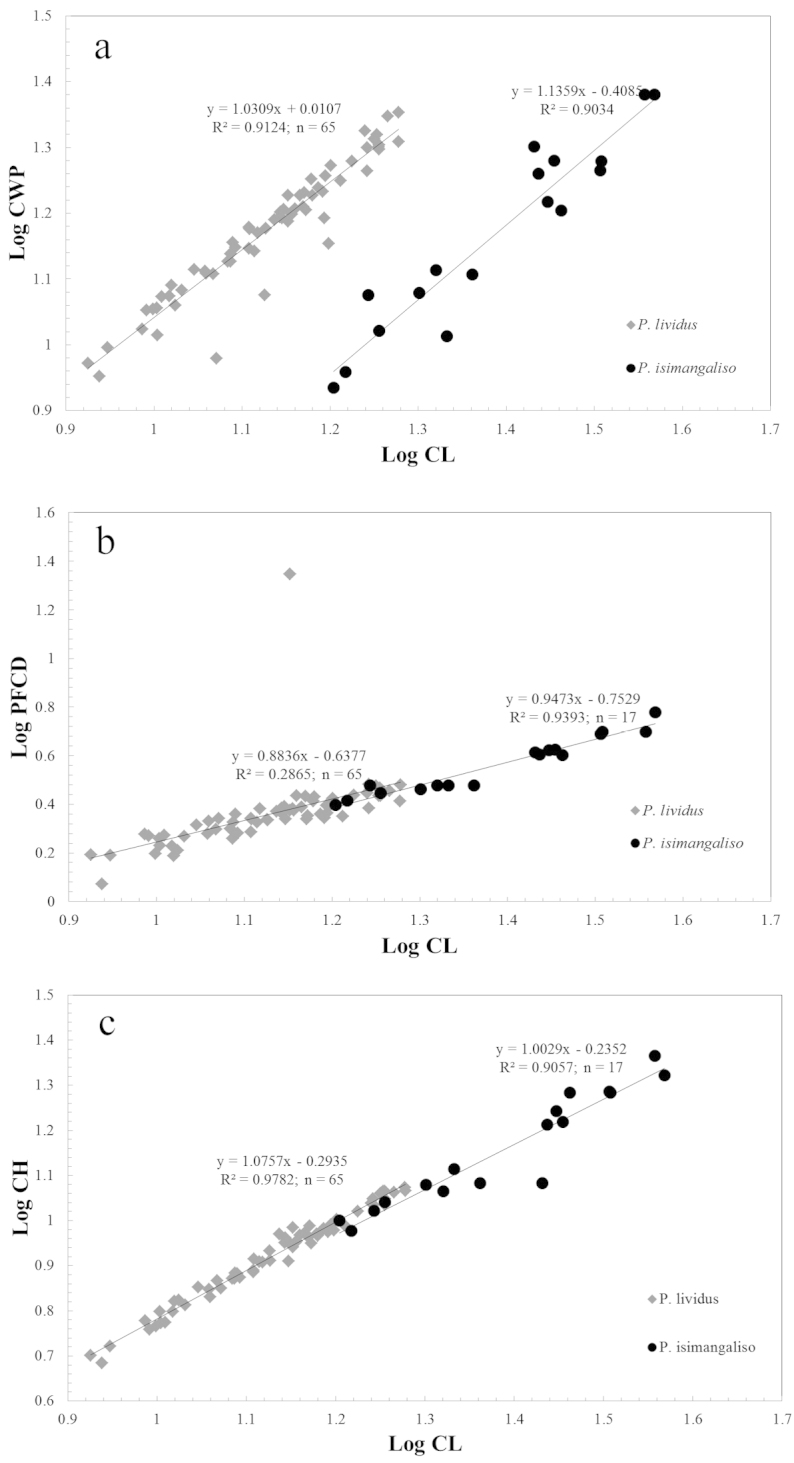
Regressions of **a** LogCWP over LogCL **b** LogPFCD over LogCL and **c** LogCH over LogCL between the two species *Potamonautes isimangaliso* and *Potamonautes lividus*. All differences between regressions were statistically significant (p < 0.001).

Individuals were reassigned to groups based on a priori probabilities using these classification functions. 100% of both forms were correctly classified with no individuals being reassigned. Three variables (CWP, PFCD and CH) were regressed over CL and a significant difference was seen between the two species as follows: CWP/CL – SS = 0.1, df = 2, F = 2.29, p < 0.001; PFCD/CL – SS = 0.1, df = 2, F = 2.29, p < 0.001; CH/CL – SS = 0.1, df = 2, F = 5.99, p < 0.001 (Fig. 3a, b, c)

## Taxonomic description

### Potamonautes isimangaliso

Taxon classificationAnimaliaDecapodaPotamonautidae

Peer & Gouws sp. n.

http://zoobank.org/4D5E76D6-BFEB-41CE-BF3D-EBD2AA3820D7

#### Type series.

Holotype: male, CL = 37 mm, ephemeral pan 200 m away from the western fence of False Bay Park (FB3), iSimangaliso Wetland Park (27°57'31.33"S, 32°21'42.15"E; elevation 62 m), 2 February 2015, R. Perissinotto, R.H. Taylor, D. Bilton, M.S. Bird, S.J. du Plooy and L. Clennell legit (ISAM A78908).

Allotype: female, CL = 27 mm, ephemeral pan, next to road leading from Dukandlovu campsite to False Bay Park entrance gate (FB5), 5 km south of Lister’s Point, iSimangaliso Wetland Park (28°0'51.70"S, 32°21'55.36"E; elevation 10 m), 1 February 2015, R. Perissinotto, R.H. Taylor, D. Bilton, M.S. Bird, S.J. du Plooy and L. Clennell legit (ISAM A78909).

Paratypes: one male, one female, collection data same as per holotype (NMMU); one male, ephemeral pan along the main road of False Bay Park (FB1), iSimangaliso Wetland Park (27°58'32.02"S, 32°21'51.62"E; elevation 42 m), 1 February 2015, R. Perissinotto, R.H. Taylor, D. Bilton, M.S. Bird, S.J. du Plooy, and L. Clennell legit (ISAM A78910); one male, ephemeral pan, collection data same as per allotype (ISAM A78911); two males, two females, ephemeral pan along the main road of False Bay Park (FB1), iSimangaliso Wetland Park (27°58'32.02"S, 32°21'51.62"E; elevation 42 m), 31 January 2015, R. Perissinotto, R.H. Taylor, D. Bilton, M.S. Bird, S.J. du Plooy, and L. Clennell legit (ISAM A78912); two females, collection data same as per holotype, 26 November 2013, R. Perissinotto, R.H. Taylor, N. Peer, N.A.F. Miranda, M.S. Bird, J.L. Raw and L. Clennell legit (NMMU); one male, one female, ephemeral pan near Sandy Point in False Bay Park (FB 6), iSimangaliso Wetland Park (27°58'36.0"S, 32°22'17.0"E; elevation 12 m), 25 November 2013, R. Perissinotto, R.H. Taylor, N. Peer, N.A.F. Miranda, M.S. Bird, J.L. Raw and L. Clennell legit (ISAM A78913); two males, ephemeral pan, collection data same as per allotype, 5 December 2012, R. Perissinotto, N.A.F. Miranda, N. Peer, J.L. Raw legit (ISAM A78914).

#### Diagnosis.

Main distinguishing features of *Potamonautes isimangaliso* from *Potamonautes lividus* Gouws, Stewart & Reavell, 2001 as follows: slightly granulated, horizontal anterolateral margin more rounded than in *Potamonautes lividus*; downward projection of postfrontal crest at exorbital edges; uniform colouration of dark purplish brown with lighter or orange coloured joints, cheliped tips and pereopods tips. *Potamonautes isimangaliso* is larger than *Potamonautes lividus*, with a maximum size of 37 mm CL recorded in males.

#### Description.

**Figure 4.**
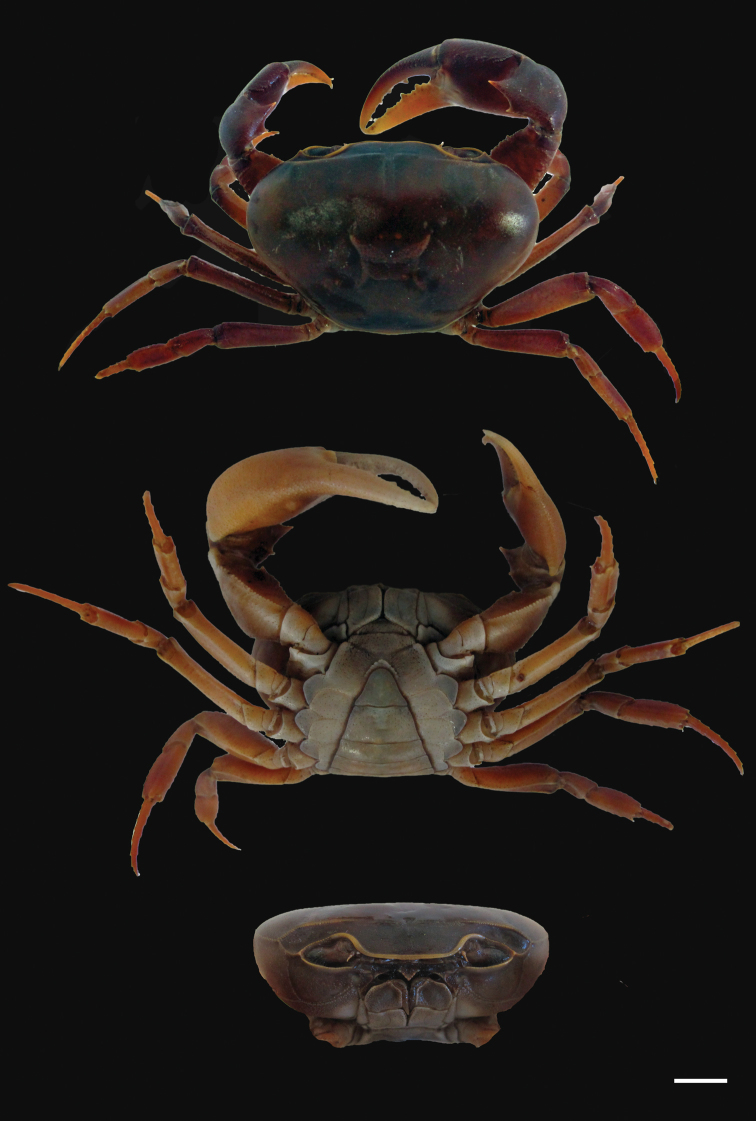
*Potamonautes isimangaliso* sp. n. male holotype CWW 55.1 mm (ISAM A78908). **a** dorsal view **b** ventral view and **c** cephalothorax, frontal aspect. Scale bar: 10 mm. Photos: Nasreen Peer.

Carapace (Fig. 4a, c). Cephalothorax somewhat vaulted (CH/CL = 0.57), wide (CWW/CL = 1.49) and ovoid in general. Branchial region extremely rounded, forming a quarter of a circle with anterolateral margin. Anterior margin straight, lying on same horizontal plane as anterolateral margin; anterolateral margin slightly granulated. Urogastric grooves well-defined; cardiac and cervical grooves well-defined where attached to the urogastric groove, but then becoming poorly defined and shallow towards edge of carapace. Epigastric lobes well-defined above postfrontal crest by two indentations forked from midpoint of postfrontal crest. Postfrontal crest slightly granulated, curving forward medially. Postfrontal crest indistinct medially but pronounced posterior to orbital margins, curving prominently downwards at epibranchial region. Moderate presence of small exorbital teeth, but complete absence of epibranchial teeth. Flank of carapace smooth, with clear horizontal (epimeral) suture separating pterygostomial region from subhepatic and suborbital regions; vertical (pleural) groove dividing subhepatic region from suborbital region.

Sternites (Fig. 4b). Sternites 1 and 2 fused; first sulcus absent as a result; second sulcus s2/s3 prominent, running completely across sternum; third sulcus s3/s4 projecting downwards medially towards abdominopelvic region. Sulci and episternal sulci thereafter well-defined but shallow.

**Figure 5.**
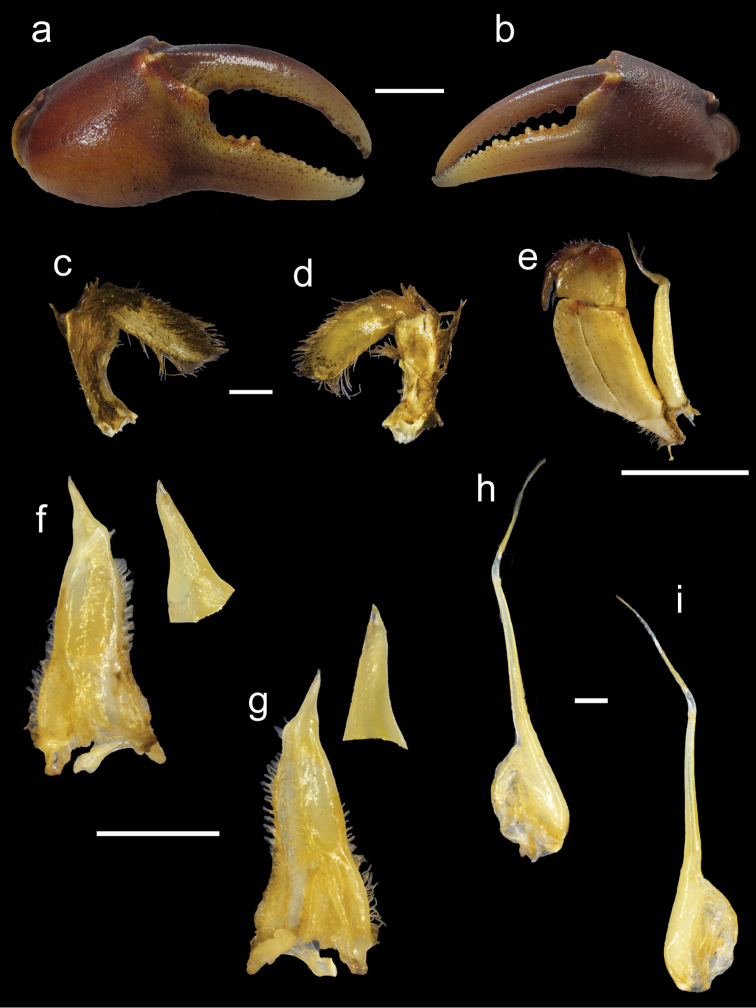
*Potamonautes isimangaliso* sp. n. male holotype CWW 55.1 mm (ISAM A78908). **a** major cheliped **b** minor cheliped **c** right mandibular palp posterior view **d** right mandibular palp anterior view **e** 3^rd^ maxilliped **f** left gonopod 1 anterior view with enlarged terminal segment **g** left gonopod 1 posterior view with enlarged terminal segment **h** left gonopod 2 anterior view and **i** left gonopod 2 posterior view. Scale bars: 10 mm (**a, b**), 1 mm (**c, d**), 10 mm (**e**), 5 mm (**f, g**), 1 mm (**h, i**). Photos: Nasreen Peer.

Third maxillipeds (Figs 4c, 5e). Filling entire buccal frame except transversely oval respiratory openings at top lateral corners. Ischium slightly scabrous, with pronounced groove running vertically. Flagellum on exopod of third maxilliped fairly long, curving upward at distal ends.

Mandibular palp (Fig. 5c, d). Consisting of two segments; terminal segment undivided and smooth, with dense tuft of setae protruding from base; hirsute margins; light covering of setae on posterior surface; subterminal segment enlarged distally where it joins with terminal segment.

Pereopods (Figs 4a, b, 5a, b). General right-handedness and prominent inequality of chelae where CRDL/CLDL = 1.32. Dactyl of major chela moderately arched; large interspace formed in major cheliped when fingers are closed, long slim interspace formed by closing of fingers in minor cheliped. Twenty four cutting teeth present on the dactyl of major cheliped and 29 on dactyl of minor cheliped; 3 larger and more prominent than the rest. Propodus fairly inflated; right propodus larger (CRPL/CLPL = 1.41) and wider (CRPW/CLPW = 1.75) than left. Left pollex with 25 cutting teeth and right propodus with 18. Carpus on either side containing one prominent tooth followed by one smaller tooth. Meri strongly granulated around margins; slender pereopods (pereopod 2: ML/MW = 3.28; pereopods 5: ML/MW = 2.17), pereopod 3 is longest and pereopod 5 shortest; ventral margins of meri smooth; ventral margins of propodi slightly serrated; dorsal margins of meri and propodi bearing fine sharp bristles; dactyli serrated and ending in sharp points.

Pleon (Fig. 4b). First five segments broad and short, with segments 6 and 7 longer; segments 1–6 four sided, with triangular distally-rounded terminal segment (telson).

Pleopods (Fig. 5f, g, h, i). Gonopod 1 bearing short terminal segment only 0.23 times the length of the subterminal segment. Terminal segment curving slightly away from midline when viewed posteriorly. Gonopod widest at base, with both subterminal and terminal segments tapering and ending in sharp point. Inner lateral margin of subterminal segment irregular; outer lateral margin curving in a concave manner towards middle of gonopod; both margins hirsute. Groove extending almost through entire length of gonopod, visible on posterior surface, lined with setae. Distal margin of subterminal segment irregularly curved. Gonopod 2 consisting of two segments; terminal segment relatively long (0.47 times length of subterminal segment), very slim; subterminal segment wide at base, sharply becoming very narrow around 0.4 of length at which point narrow process forms, leading up to terminal segment. Small tuft of setae present on outer margin of base of subterminal segment. Gonopod 2 curved, moving outwards away from medial line of gonopod proximally, curving back towards medial line distally.

Variation. The major cheliped does not always display a pronounced interspace when fingers are closed. In juveniles and in the female allotype this was less prominent. The arching of chelipeds varies too, with some (particularly the minor chelipeds) bearing straight dactyli while others are fairly rounded. All collected specimens display a pronounced heterochely and all appear to be right-handed.

**Table 1.** Morphometric variables (mm) of *Potamonautes isimangaliso* sp. n. holotype and paratype specimens.

Variable	Holotype	Males (n=8)	Females (n=7)
CL	37	13.2–36.1	18–27
CWW	55.1	18–53	26–40.1
CPW	24	9–24	10.5–20
PFCD	6	2–5	2.8–4.1
ED	15.6	5–16	8.5–12.8
CWA	34	14.1–40.5	21.5–32
CH	21	7–23.2	11–12.1
AW6	12	3.1–11	7–23.4
MCPL	49.3	11.5–44.5	17.2–29.2
MCPH	21.9	4.1–20.1	7–13.7
P2ML	21.3	6.5–18.9	8.9–13.8
P2MH	6.5	2–6.1	3–5

#### Live colouration.

Colouration of carapace may vary between light brown, maroon, purplish-brown and almost black. Similarly, tips of dactyli may be either orange, bright yellow or a dull yellow.

#### Distribution.

Currently only known from the False Bay region of the iSimangaliso Wetland Park on the north-east coast of South Africa.

#### Type locality.

South Africa, KwaZulu-Natal, iSimangaliso Wetland Park, False Bay - Western Shores: Mpophomeni Pan (27°57'31.33"S, 32°21'42.15"E); Dukandlovu Pan (28°0'51.70"S, 32°21'55.36"E); Main Road Pan (27°58'32.02"S, 32°21'51.62"E); Sandy Point Pan (27°58'36.0"S, 32°22'17.0"E).

#### Etymology.

The species is named after the iSimangaliso Wetland Park, located in northern KwaZulu-Natal, where it is currently thought to be micro-endemic. This is significant as the iSimangaliso Wetland Park falls within the Maputaland centre of endemism (Smith et al. 2008), highlighting the importance of this park as a global biodiversity hotspot. The Park is a UNESCO World Heritage Site and contains three Ramsar Wetlands of International Importance.

#### Remarks.

*Potamonautes isimangaliso* sp. n. is easily distinguishable from most other *Potamonautes* spp. found in South Africa. *Potamonautes dentatus* Stewart, Coke & Cook, 1995, *Potamonautes parvispina* Stewart, 1997, *Potamonautes unispinus* Stewart & Cook, 1998, *Potamonautes warreni* Calman, 1918 and *Potamonautes calcaratus* (Gordon, 1929) all bear dentate anterolateral margins or epibranchial corners, while *Potamonautes isimangaliso* has a rounded epibranchial corner and mildly granular anterolateral margin.

*Potamonautes perlatus* (H. Milne Edwards, 1837), *Potamonautes granularis* Daniels, Stewart & Gibbons, 1998, *Potamonautes sidneyi* Rathbun, 1904, *Potamonautes barbarai* Phiri & Daniels, 2014 and *Potamonautes barnardi* Phiri & Daniels, 2014 all have sharply-defined scabrous or granular epibranchial corners and prominent postfrontal crest, while *Potamonautes isimangaliso* has a heavily rounded smooth epibranchial corner and poorly-defined postfrontal crest. *Potamonautes parvicorpus* Daniels, Stewart & Burmeister, 2001 also displays a finely granulated anterolateral margin and rounded epibranchial corners, but the resemblance to *Potamonautes isimangaliso* is superficial, as it differs in the indentation of its anterior margin where *Potamonautes parvicorpus* bears a slightly indented anterior margin while that of *Potamonautes isimangaliso* lies straight. Further differences are seen in locality as the habitat of *Potamonautes parvicorpus* is restricted to high mountain streams in the Western Cape (Daniels et al. 2001).

*Potamonautes clarus* Gouws, Stewart & Coke, 2000, *Potamonautes depressus* (Krauss, 1843), *Potamonautes brincki* (Bott, 1960), *Potamonautes flavusjo* Daniels, Phiri & Bayliss, 2014 and *Potamonautes lividus* Gouws, Stewart & Reavell, 2001 all have smooth anterolateral margins and rounded smooth epibranchial corners but bear differences compared to *Potamonautes isimangaliso*. One of the diagnostic characters of *Potamonautes depressus* is the dorsally flattened carapace, where CL/CH = 2.3–2.6. *Potamonautes isimangaliso* has a more vaulted carapace with a CL/CH ratio of 1.6–1.8. *Potamonautes brincki* and *Potamonautes clarus* are smaller crabs (max CL = 27 mm in males for both species), preferring fast-flowing mountain stream habitats. *Potamonautes flavusjo* is ecologically distinct from *Potamonautes isimangaliso* and can be found in the Mpumalanga Highveld. In addition to this, the species is smaller and has flattened chelipeds, not adapted for burrowing (Daniels et al. 2014). Light yellow chelipeds and ventral surfaces of pereopods characterise *Potamonautes flavusjo*.

*Potamonautes lividus* shares a similar distribution, outward appearance and preference for air-breathing with *Potamonautes isimangaliso*. However various differences exist between the two species.The level and angle of anterolateral margin differ, where *Potamonautes isimangaliso* bears an anterolateral margin lying on the same horizontal plane as the anterior margin. Conversely, *Potamonautes lividus* has an anterolateral margin which angles downward to join the anterior margin and thus sits higher than the anterior margin. The downward angle of postfrontal crest at exorbital edges is seen in *Potamonautes isimangaliso* but not in *Potamonautes lividus*. Carapace flatness is indicated by the CL/CH ratio which equates to 1.5 for *Potamonautes lividus* and 1.8 for *Potamonautes isimangaliso* holotypes. The maximum size (37 mm CL in *Potamonautes isimangaliso* and 25.5 mm CL in *Potamonautes lividus*), colouration (dark blue carapace with bright orange chelipeds in *Potamonautes lividus* and dark brown/purple carapace with brown or dull yellow cheliped in *Potamonautes isimangaliso*), inflation of chela with gap between propodus and dactyl (dactyl of *Potamonautes lividus* is more arched than that of *Potamonautes isimangaliso*) and the number of poorly-developed teeth on carpus (*Potamonautes lividus* containing one prominent and three rudimentary teeth; *Potamonautes isimangaliso* containing one prominent and one rudimentary tooth) further distinguish the two species. Gonopods of both species are very similar with the only difference being the tuft of setae found at the base of pleopod 2 in *Potamonautes isimangaliso*. Specimens resembling *Potamonautes lividus* were found in the Dwesa Forest, Eastern Cape and appear to be genetically nearly identical to *Potamonautes lividus* (Daniels et al. 2014). This further substantiates the genetic distinctiveness of *Potamonautes isimangaliso*. The smallest mature male of *Potamonautes isimangaliso* recorded had a CL of 13.2 mm whilst all females recorded were mature (min CL = 18 mm).

Preliminary sequence data for two mitochondrial gene regions (16S: GenBank accession number KR137640; COI: KR137642) generated from a representative of the new species, using approaches described elsewhere (Daniels et al. 2002; Phiri and Daniels 2014; G. Gouws unpubl.), were notably divergent (7.3% and 7.9%, respectively) from published 16S (AY042248; Daniels et al. 2002) and COI (AF510879; Daniels et al. 2002) sequences of *Potamonautes lividus* from KwaZulu-Natal.

#### Habitat and ecology.

**Figure 6.**
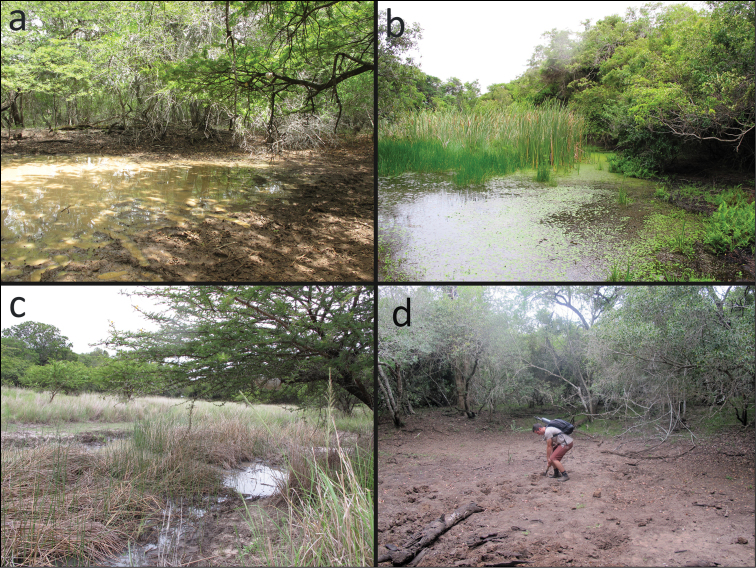
Sampling localities of *Potamonautes isimangaliso* sp. n.: **a** Main Road Pan (FB1) **b** Mpophomeni Pan (FB3) **c** Dukandlovu Pan (FB5) and **d** Sandy Point Pan (FB6), completely dry during Feb 2015. Photos: **a–c** Lynette Clennell; **d** Nasreen Peer.

*Potamonautes isimangaliso* sp. n. inhabits freshwater ephemeral pans (maximum salinity recorded: 0.75) which fill up with fresh, oxygen-deprived water after rainfall events, mainly during the summer wet season. These pans are located along the western shores of False Bay, Lake St Lucia (Fig. 6a–d) in clearings of the sand forest biome of False Bay and are generally partially shaded. *Potamonautes isimangaliso* and *Potamonautes lividus* are found in close proximity although *Potamonautes lividus* has not been found in False Bay Park and *Potamonautes isimangaliso* has not been found outside of the park. Furthermore, a difference in habitat type between *Potamonautes isimangaliso* and *Potamonautes lividus* Gouws, Stewart & Reavell, 2001 is seen, where the latter is known to inhabit burrows well above the surface water line in *Ficus* and *Barringtonia* swamps (Gouws et al. 2001), while the new species was found in close association with ephemeral pans in sand forest habitat with burrows extending below the surface waterline. Vegetation types include the dominant canopy species *Cleisthantus schlechteri*, *Hymenocardia ulmoides*, *Psydrax fragrantissima*, *Croton pseudopulchellus* and *Drypetes arguta* (Kirkwood & Midgley, 1999), as well as various *Acacia* spp. (Moll 1980). Grass species such as *Paspalum vaginatum* and *Eleochoris* sp. are also closely associated with this environment (Moll 1980). Aquatic plants associated with the ephemeral pans include the reed *Typha capensis*, the sedge *Juncus kraussii*, the mangrove fern *Acrostichum aureum* and the duckweed *Lemna* sp. (Howard-Williams 1980).

**Figure 7.**
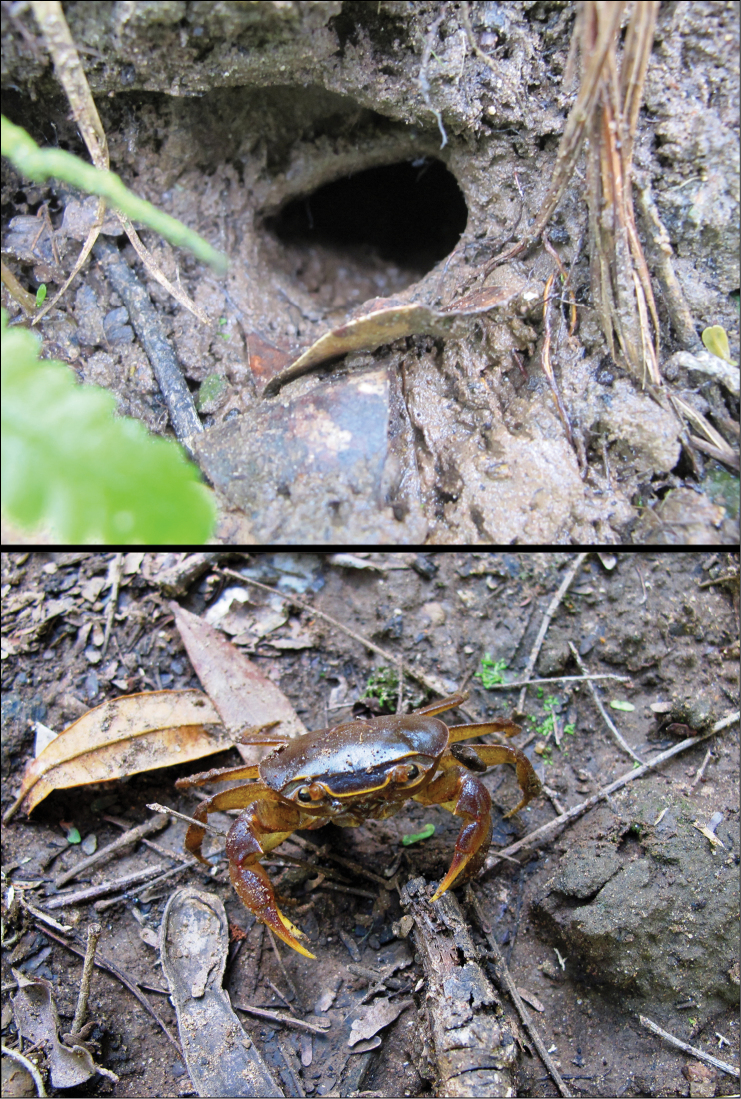
**A** Burrows of *Potamonautes isimangaliso* sp. n. are typically found on the banks of ephemeral pans and are even maintained when pans are completely dry **B** *Potamonautes isimangaliso* sp. n. in its natural habitat. Photos: Lynette Clennell.

*Potamonautes isimangaliso* adults form burrows on the banks of these pans (Fig. 7a), while juveniles are found either in burrows or free-crawling in shallow water (2–50 cm). The species lives sympatrically with *Potamonautes sidneyi* but is separated by habitat type, with *Potamonautes sidneyi* inhabiting flowing streams and able to withstand higher salinities of up to 9 (18 May 2013, Mpophomeni Stream, 27°57'7.17"S, 32°22'37.21"E). Oxygen levels in the pans inhabited by *Potamonautes isimangaliso* are quite low compared to flowing streams (Table 2).

**Table 2.** Physico-chemical parameters of sampling localities †.

	Main Road Pan (FB1)	Mpophomeni Stream (FB2)	Mpophomeni Pan (FB3)	Dukandlovu Pan (FB5)
Temperature (°C)	26.1	20.4	22.03	26.73
Salinity	0.15	8.36	0.29	0.75
Maximum depth (mm)	80	500	700	250
pH	7.2	7.03	7.42	6.9
Turbidity (NTU)	1310.5	14	151	306.3
Dissolved oxygen (% sat.)	19.8	90.1	22.4	69.6

† No data is included for Sandy Point Pan (FB6) as the site was dry at the time of sampling.

Although the species appears to be more closely associated with water than its morphologically closest congener, *Potamonautes lividus* (Gouws et al. 2001), the low levels of oxygen characteristic of the pans along with the ephemeral nature of the waterbodies indicate a greater affinity for a terrestrial lifestyle, as it may be more effective to obtain oxygen through air-breathing. This has been recorded previously in various African freshwater brachyuran genera and a high-vaulted carapace may be indicative of this change, where periods of dryness favour the evolution of burrowing semi-terrestrial, air-breathing tendencies (Cumberlidge 1999; Cumberlidge 2009). Specimens of *Potamonautes isimangaliso* have been observed in deep burrows (30–50 cm) around desiccated pools. Because the rainy season in this area is generally restricted to the period November-April (late Austral spring to early Autumn), much of the population hibernates deep in the mud, where traces of moisture persist throughout the dry season. Crabs return to the surface only after major rainfall events have filled up the ephemeral pools. The summer of 2014–2015 had been particularly dry in the area, with substantial rain falling in the False Bay area starting only in the middle of January (69 mm during 15–17 Jan, 54 mm during 28–30 Jan 2015; False Bay Park Meteo Station). Numerous adult and sub-adult crabs were observed from 31 Jan to 3 Feb in the newly filled ephemeral pools but hardly any young juvenile, indicating that the previous drought conditions had not allowed spawning to happen yet.

The feeding ecology of the species is largely unknown, although *Potamonautes* crabs are generally thought to shift from a diet of aquatic invertebrates to a more herbivorous or opportunistic diet with age (Hill and O’Keeffe 1992). The chelar dentition is serrate and the larger crusher chela lacks rounded or molariform occlusive geometry in the proximal region, probably due to wearing down over time. The dentition of the chela is indicative of an opportunistic omnivorous diet (Yamada and Boulding 1998).

### Updated key for the identification of the *Potamonautes* species of South Africa

**Based on Day et al. (2001)**

**Table:** 

1	Anterolateral margin bearing one tooth or many distinct teeth	**2**
–	Anterolateral margin smooth tooth, sometimes serrated or granulated	**6**
2	Anterolateral margin bearing two or more distinct teeth	**3**
–	Anterolateral margin bearing one distinct tooth at epibranchial corner	**4**
3	Postfrontal crest complete to anterolateral margin; epibranchial sinus absent	***Potamonautes warreni* Calman, 1918**
–	Postfrontal crest interrupted at exorbital teeth; epibranchial sinus present	***Potamonautes dentatus* Stewart, Coke & Cook, 1995**
4	Postfrontal crest not continuous posterior to exorbital teeth; merus of chelipeds bearing a spine on both antero- and posterior-inferior granulate margins	***Potamonautes calcaratus* (Gordon, 1992)**
–	Postfrontal crest complete; no spine on merus of cheliped	**5**
5	Postfrontal crest not sloping backwards towards anterolateral margin; epibranchial sinus absent	***Potamonautes unispinus* Stewart & Cook, 1998**
–	Postfrontal crest sloping backwards to meet anterolateral margin; epibranchial sinus present	***Potamonautes parvispina* Stewart, 1997**
6	Anterolateral margin granulated, forming distinct angle with postfrontal crest at epibranchial corner; epibranchial region usually scabrous or granulated	**7**
–	Anterolateral margin rounded and smooth, meeting postfrontal crest at rounded epibranchial corner; epibranchial region usually without scabrosity or granulation	**10**
7	Carapace and postfrontal crest strongly granulated, with pronounced scabrosity in epibranchial region	**8**
–	Carapace and postfrontal crest moderately granulated, with relatively smooth epibranchial region	**9**
8	Carapace anterior relatively narrow and curved moderately over the branchial region; inward-extending lobe absent from short terminal segment of gonopod 2; confined to the Olifants River system in the Cape Fold Mountains below the Bulshoek dam wall (WC)	***Potamonautes granularis* Daniels, Stewart & Gibbons, 1998**
–	Carapace anterior relatively wide and curved slightly over the branchial region; long slender S-shaped terminal segment of gonopod 2 is formed by inward extending lobe; not occurring in the Western Cape	***Potamonautes sidneyi* Rathbun, 1904**
9	Occurring largely in the Western Cape, also extending further north and east; found in western flowing drainage systems including the Olifants River, where it occurs above the Bulshoek dam wall	***Potamonautes perlatus* (H. Milne Edwards, 1837)**
–	Restricted to southern flowing drainages in the Western Cape (Gamtoos River and Gourits River)	***Potamonautes barbarai* Phiri & Daniels, 2014**
–	Restricted to the Berg River and tributaries of the Breede River (WC)	***Potamonautes barnardi* Phiri & Daniels, 2014**
10	Carapace depressed and dorso-ventrally flattened; ratio of carapace length to height between 2.1 and 2.6	**11**
–	Carapace vaulted and arched; ratio of carapace length to height between 1.5 and 2.2	**12**
11	Carapace flatter, with a ratio of carapace length to carapace height between 2.3 and 2.6; postfrontal crest often directed forward near anterolateral margin; dark yellow-brown or green-brown in colour	***Potamonautes depressus* (Krauss, 1843)**
–	Carapace more vaulted, with ratio of carapace length to carapace height between 2.1 and 2.3; postfrontal crest straight near the anterolateral margin; orange in colour	***Potamonautes clarus* Gouws, Stewart & Coke, 1995**
12	Dactyli of chelipeds flattened; postfrontal crest, pereopods and chelipeds bright yellow; occurring in the Mpumalanga Highveld	***Potamonautes flavusjo* Daniels, Phiri & Bayliss, 2014**
–	Dactyl of chelipeds moderately or highly arched	**13**
13	Anterolateral margin curving inward over carapace surface in the branchial region; occurring in KZN	**14**
–	Anterolateral margin not curving inward over carapace surface in the branchial region; occurring in the WC	**15**
14	Bearing one prominent tooth and three rudimentary teeth on carpus of cheliped; tuft of setae absent from base of pleopod 2; carapace dark with distinctive blue sheen; chelipeds and pereopods bright orange	***Potamonautes lividus* Gouws, Stewart & Reavell, 2001**
–	Bearing one prominent tooth and one rudimentary tooth on carpus of cheliped; tuft of setae present at base of pleopod 2; carapace uniformly purplish brown with similar coloured or slightly brighter chelipeds and pereopods	***Potamonautes isimangaliso* sp. n.**
15	Flange present on terminal segment of mandibular palp	***Potamonautes brincki* (Bott, 1960)**
–	Flange absent from terminal segment of mandibular palp	***Potamonautes parvicorpus* Daniels, Stewart & Burmeister, 2001**

## Supplementary Material

XML Treatment for Potamonautes isimangaliso
